# Systematic review of clinical literature for absent common carotid artery

**DOI:** 10.1016/j.jvscit.2025.102039

**Published:** 2025-11-12

**Authors:** Wade Hopper, Hunter Row, Carter West, Ossama Reslan

**Affiliations:** a Department of Surgery, University of North Dakota School of Medicine and Health Sciences, Fargo, ND; b Department of Vascular Surgery, Fargo Veterans Affairs Medical Center, Fargo, ND

**Keywords:** Absent common carotid artery, Cardiovascular abnormalities, Carotid stenosis, Case report, Systematic review

## Abstract

**Objective:**

Absent common carotid artery (ACCA) is a rare condition, with less than 125 cases reported in the literature. Trends in symptoms, treatments, and outcomes related to ACCA are largely unknown.

**Methods:**

We present a case of ACCA in a 77-year-old man who also had asymptomatic severe atherosclerotic stenosis of the ipsilateral internal carotid artery (ICA). Carotid revascularization was performed via transposition of the ipsilateral internal to external carotid artery. The case was successful, and he recovered without complication. We also performed a systematic review of EMBASE and PubMed databases for clinical presentations and treatment outcomes of ACCA. Literature quality was assessed according to the Joanna Briggs Institute case report checklist.

**Results:**

We identified 64 cases of ACCA from 61 reports. Over 50% of patients with ACCA presented with cerebrovascular-related symptoms, including 21 (33%) presenting acutely with transient ischemia attack or cerebrovascular accident. Incidence of intracranial aneurysm was disproportionately high, occurring in 10 cases (16%). Hypoplasia or absence of the ipsilateral ICA was seen in 22 cases (35%). Treatment for carotid stenosis or intracranial aneurysm was reported in 12 cases (20%), with no reports of complication or mortality. Only four reports (7%) documented patient race or ethnicity, and 19 (31%) did not describe clinical management.

**Conclusions:**

We recommend diagnostic head imaging to detect intracranial aneurysm in adult patients found to have ACCA and hypoplasia or tortuosity of the ipsilateral ICA. We also recommend carotid revascularization in patients found to have severe stenosis of the ipsilateral ICA, and this can safely be performed with open or endovascular methods. Current ACCA case report literature is of low quality and would benefit from improved reporting of patient demographics and clinical management strategies.

Absence of the common carotid artery (ACCA) is a rare congenital vascular anomaly, with fewer than 125 cases reported in the literature to date including cadaveric studies.^1^^,^^2^ In this condition, the common carotid artery (CCA) fails to form, and the internal carotid artery (ICA) and external carotid artery (ECA) arise independently, most commonly from the aortic arch or the brachiocephalic artery. However, a wide spectrum of anatomic variants have been described.^1^ The embryologic basis of ACCA is believed to involve persistence of the *ductus caroticus*, a transient embryonic structure connecting the third and fourth primitive dorsal aortae that normally regresses by the sixth week of gestation.^1^ Its failure to involute is thought to disrupt the typical fusion and development of the CCA. Two systematic reviews have previously addressed this anomaly. In 2019, Vasovic et al provided a comprehensive analysis of anatomic and embryologic variations associated with ACCA, offering a foundational reference for morphologic classification.^1^ A more recent review expanded the total number of reported cases to 123, including cadaveric findings, but did not explore presenting symptoms, clinical management, or treatment outcomes in detail.^2^ Given this gap in the literature, we conducted a systematic review focused on the clinical presentation, diagnostic approach, management strategies, and outcomes of patients with ACCA. Additionally, we report a novel case of right-sided ACCA associated with hemodynamically significant stenosis of the ICA and successful treatment with open surgical transposition of the ICA to the ECA.

## Methods

### Case report

We present the case of a patient diagnosed and treated at our institution between October 2024 to July 2025 for severe stenosis of a right ICA independently originating from the brachiocephalic artery in the setting of right-sided ACCA. A retrospective review of the electronic medical record was conducted to obtain all relevant clinical documentation and imaging related to the course of care. Informed consent for publication was obtained from the patient.

### Systematic review

**Fig 1:**
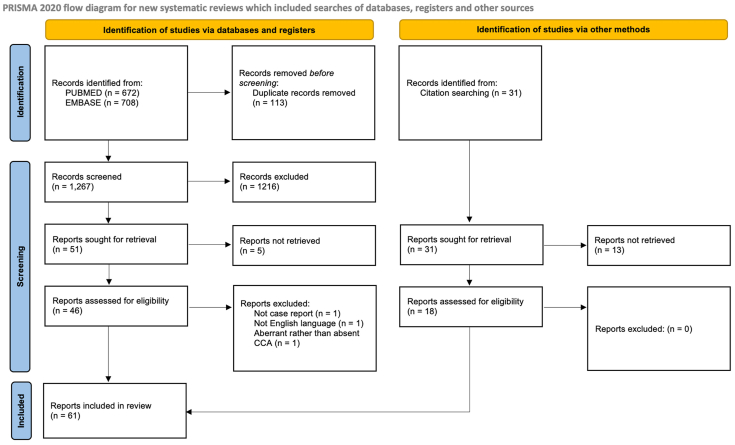
Systematic review flowchart and search results. Rayyan software was used to identify duplicate records which were then manually assessed by authors for exclusion. *PRISMA*, Preferred Reporting Items for Systematic Reviews and Meta-Analyses.

We conducted an unregistered systematic review of the literature using the PubMed and EMBASE databases from January 1, 1900, up to July 8, 2025. The search strategy employed was: “common carotid artery” AND (“independent origin” OR “absence” OR “absent” OR “aberrancy” OR “aberrant” OR “aplasia” OR “agenesis”). Eligibility criteria for further screening were English-language clinical literature describing absence of the common carotid artery and documentation of presenting symptoms. Exclusion criteria included cadaveric studies. Titles and abstracts were independently screened by two reviewers (W.H., C.W.). Full-text review was then performed for studies meeting inclusion criteria. Additional sources were identified through reference and citation tracking of selected articles. Duplicate records were identified by Rayyan web-based software. Study authors (W.H., C.W.) directly screened and removed duplicates. No generative artificial intelligence tools were used during the review process. Demographic, clinical, anatomic, and treatment data were extracted from each included report and summarized in tabular format by one author (W.H.). Missing variables were left blank. The Joanna Briggs Institute (JBI) 8-point checklist for case reports^3^ was used to independently assess quality and bias for each included manuscript (W.H., C.W.). A JBI score of 7 or 8 was considered high quality. We adhered to the Preferred Reporting Items for Systematic Reviews and Meta-Analyses (PRISMA) 2020 statement^4^ to enhance reporting quality (Supplementary Table I, online only). A PRISMA flow chart detailing the study selection process is available in Fig 1.

## Results

### Case report

**Fig 2:**
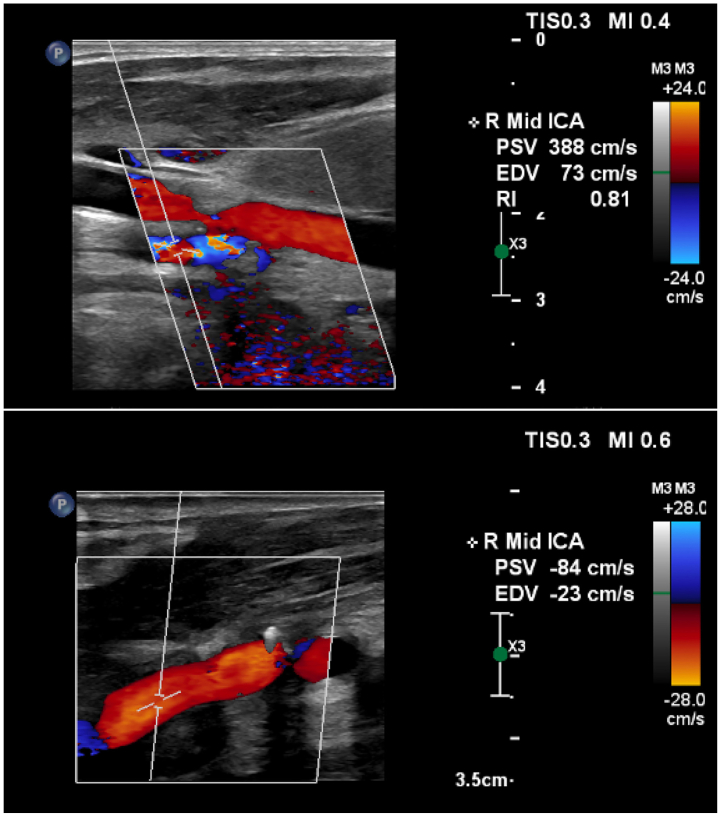
Carotid arterial duplex ultrasonography. Preoperative (*top*) right carotid artery duplex ultrasonography demonstrated preoperative flow velocities consistent with severe stenosis; follow-up imaging performed 1 month postoperatively demonstrated a widely patent right internal carotid artery (*ICA*) with no appreciable stenosis or atherosclerotic burden.

A 77-year-old man was referred to vascular surgery after screening carotid duplex demonstrated severe right ICA stenosis with a peak systolic velocity of 388 cm/s (Fig 2). He was asymptomatic, with no history of focal neurologic symptoms. Past medical history included hypertension, dyslipidemia, severe chronic obstructive pulmonary disease on home oxygen, and 40-pack-year-history of smoking tobacco use. He was independent in activities of daily living and had no prior neck or chest surgery. Clinical examination was unremarkable.

**Fig 3:**
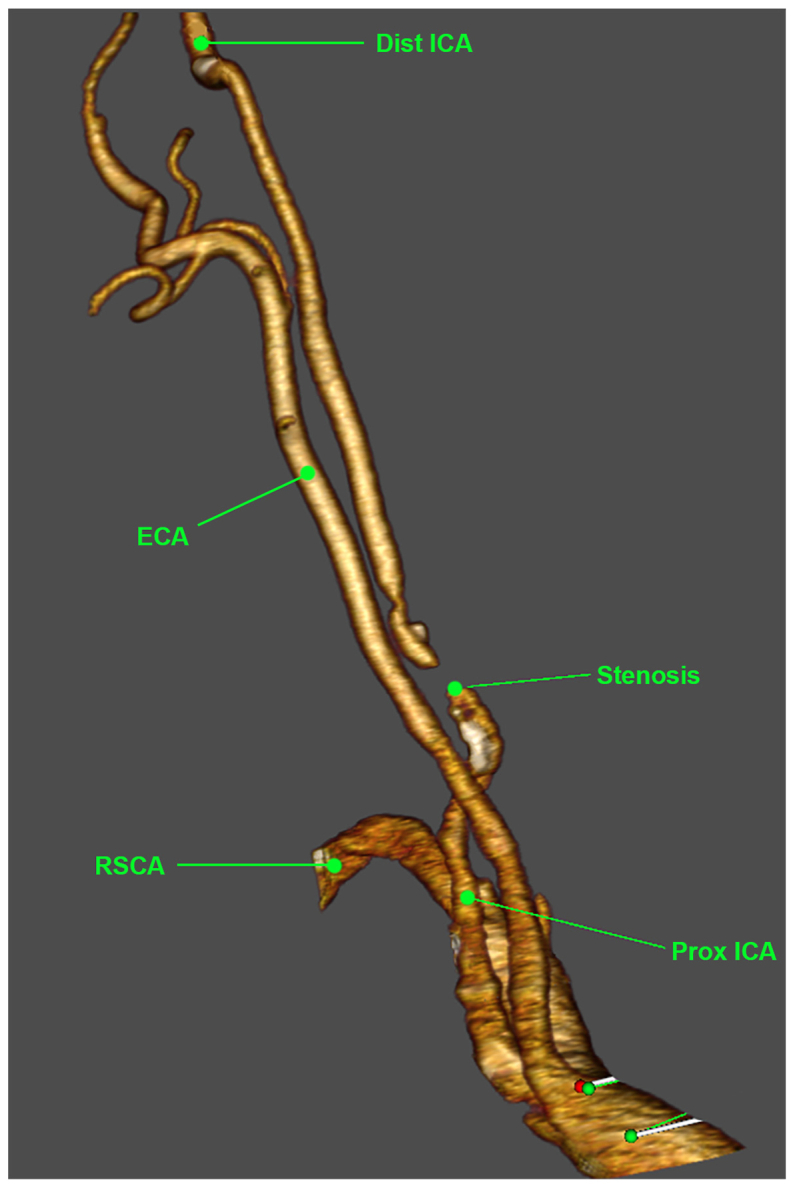
Preoperative three-dimensional chest computed tomography angiography. Frontal view of three-dimensional reconstruction of preoperative chest computed tomography angiography is shown with relevant structures labeled. The area of stenosis was approximately 3 to 4 cm from the anomalous origin of the right internal carotid artery (*ICA*) at the brachiocephalic artery. *Dist ICA*, Distal internal carotid artery; *ECA*, external carotid artery; *Prox ICA*, proximal internal carotid artery; *RSCA*, right subclavian artery.

**Supplementary Fig 1 (online only):**
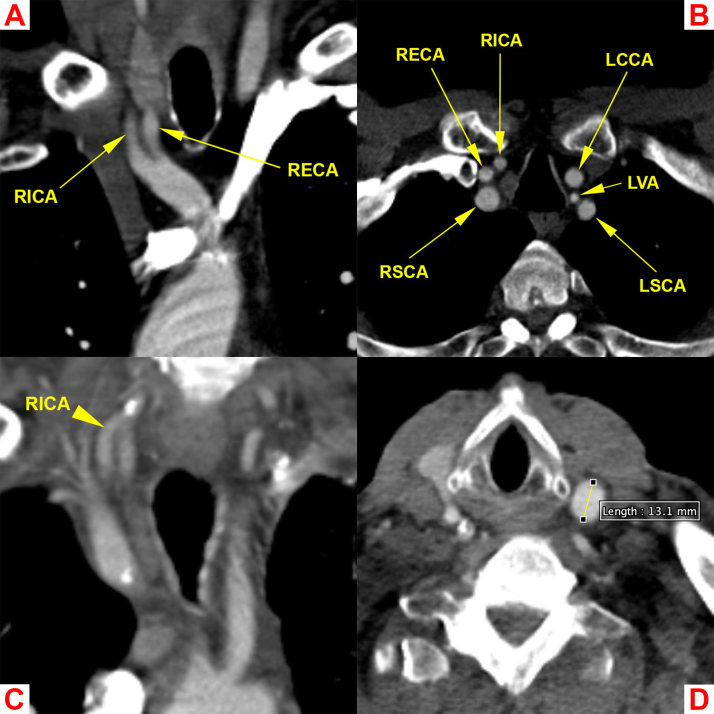


Computed tomography angiography was obtained, which showed independent origins of the right ICA and ECA originating from the brachiocephalic artery (Fig 3; additional images shown in Supplementary Fig 1, online only). Severe ICA stenosis (90% by North American Symptomatic Carotid Artery Trial criteria) was 3 cm from the aortic takeoff. The left CCA was aneurysmal (1.3 cm), and the left vertebral artery originated directly from the aortic arch. After discussion, the patient elected to undergo surgical revascularization.

**Fig 4:**
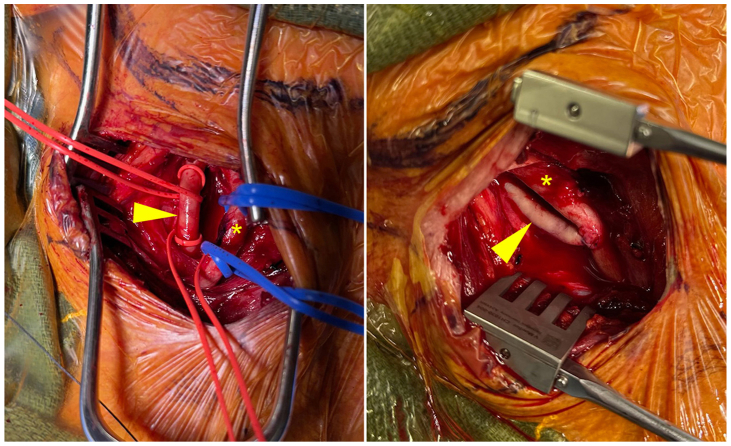
Intraoperative anatomy. A right longitudinal cervical incision was made, and carotid vasculature is pictured both before (*left*) and after (*right*) transposition of the internal carotid artery (ICA; *arrow*) to the external carotid artery (ECA; *star*).

He underwent elective transposition of the right ICA to ECA under general anesthesia with near-infrared spectroscopy neuromonitoring. Through a right neck incision, the ICA and ECA were dissected and the surrounding neurovascular structures preserved (Fig 4). After systemic heparinization, angiography confirmed anatomy. The right ICA was transected, its proximal stump oversewn, and the distal right ICA was anastomosed end-to-side to the right ECA. Intraoperative pulses were strong, and neuromonitoring remained stable.

The patient had an uncomplicated recovery and was discharged home on postoperative day 2 with aspirin and clopidogrel dual antiplatelet therapy. At 1-month follow-up, duplex ultrasound showed normal ICA velocities. No wound complications or neurologic deficits were noted.

### Systematic literature review

Searches of PubMed and EMBASE databases returned 1380 articles. After removal of 113 duplicates, 51 records remained. An additional 38 reports were identified through reference review, yielding 81 records for full-text screening. Of these, 28 were excluded due to inaccessible documents or insufficient clinical details. A total of 61 studies met inclusion criteria and were included in the final analysis.^2^^,^^5^, ^6^, ^7^, ^8^, ^9^, ^10^, ^11^, ^12^, ^13^, ^14^, ^15^, ^16^, ^17^, ^18^, ^19^, ^20^, ^21^, ^22^, ^23^, ^24^, ^25^^,^^26^, ^27^, ^28^, ^29^, ^30^, ^31^, ^32^, ^33^, ^34^, ^35^, ^36^, ^37^, ^38^, ^39^, ^40^, ^41^, ^42^, ^43^, ^44^, ^45^^,^^46^, ^47^, ^48^, ^49^, ^50^, ^51^, ^52^, ^53^, ^54^, ^55^, ^56^, ^57^, ^58^, ^59^, ^60^, ^61^, ^62^, ^63^, ^64^ All data extracted from records are presented in Supplementary Table II (online only).

Summary data for patients identified through literature review is presented in the Table. Across 63 patients, 13 (21%) were pediatric and 12 (19%) were over 65 years old. Females represented 56% of the cohort. ACCA was left-sided in 33 cases (53%) and bilateral in four cases (6%). The ipsilateral ICA was absent in four cases (6%) and hypoplastic in 18 cases (29%); eight were described as tortuous (13%). Ipsilateral ICA atherosclerosis was documented in eight patients (13%), including four with severe stenosis (6%). Of the nine patients with clinically evident stenosis (including our own case), four had associated arterial hypoplasia or tortuosity. Aneurysmal cerebrovascular disease was seen in 10 patients (16%), with eight such cases also having hypoplastic or absent ipsilateral ICA. Among aneurysm cases, 70% were female, and 60% presented with acute stroke.

**Table:** Summary of literature review

Characteristic	No. patients (N = 63)	%
Patient characteristics		
Age, years		
<18	13	21
18-65	38	60
>65	12	19
Sex		
Female	35	56
Male	26	41
Not reported	2	3
ACCA laterality		
Left	33	53
Right	26	41
Bilateral	4	6
Ipsilateral ICA absent	4	6
Ipsilateral ICA stenosis	8	13
Ipsilateral ICA hypoplasia	18	29
Ipsilateral ICA tortuosity	8	13
Cervical aortic arch	5	8
Cerebrovascular aneurysm	10	16
Presenting symptoms		
Cerebrovascular accident	11	17
Transient ischemic attack	10	16
Headache	5	8
Vertigo	4	6
Pulsatile neck mass	6	10
Congenital heart disease workup	6	10
Steal syndrome	2	3
Carotid screening	2	3
Incidental/other	17	27
Cerebrovascular treatment		
Total	12	19
Carotid revascularization	6	10
Open	3	5
Endovascular	3	5
Complication	0	0
Mortality	0	0
Intracranial aneurysm	6	10
Craniotomy	4	6
Endovascular	2	3
Complication	0	0
Mortality	0	0

*ACCA,* Absent common carotid artery; *ICA,* internal carotid artery.

Demographic and anatomic characteristics, chief symptom at presentation, and outcomes of operative treatment are reported for all patients with ACCA identified through systematic literature review. Cerebrovascular-related symptoms with low incidence were classified as other. Incidental findings were defined as those unrelated to cerebrovascular disease. Surgical indications were for atherosclerotic or aneurysmal disease.

Acute cerebrovascular accident (CVA) or transient ischemic attack (TIA) occurred in 33% of patients. An additional nine patients (14%) presented with cerebrovascular symptoms such as headache or vertigo. Two patients (3%) were diagnosed through routine carotid screening. Clinically evident aortic pathology was present in six patients (10%), including one Stanford type A aortic dissection and five cervical aortic arches presenting as pulsatile neck masses. Another pulsatile neck mass case was attributed to carotid aneurysmal disease. Congenital heart disease workup revealed ACCA in six patients (10%). Seventeen patients (27%) were diagnosed incidentally.

Operative treatment for cerebrovascular disease was reported in 12 patients (19%), all adults. Open ipsilateral ICA surgery was performed in three cases (5%): Two underwent aneurysm resection with end-to-end anastomosis, and one had ICA-to-ECA transposition for vertebral steal syndrome. Carotid stenting was performed in three cases (5%), two involving the ipsilateral ICA and one contralateral. Intracranial aneurysm treatment was documented in six patients (10%), including four craniotomies, three of which involved aneurysmal clipping.

Overall quality of reported literature was low, with a median JBI checklist score of 4 (interquartile range, 5-7) of a possible 8 points (Supplementary Table III, online only). There were 21 articles (34%) considered high quality with JBI checklist score of 7 or 8. The demographic distribution of ACCA remains incompletely known because only four reports (7%) explicitly stated patient race or ethnicity. Clinical management strategy was unclear or not described in 19 articles (31%).

## Discussion

Our systematic review revealed that approximately one-half of patients with absence of the CCA presented with cerebrovascular-related symptoms, including 33% with CVA or TIA. Additionally, 35% of patients had a hypoplastic or absent ipsilateral ICA, with a strong association between abnormal ICA morphology and intracranial aneurysms. This aligns with prior literature on hypoplastic ICA, which is associated with increased aneurysm formation due to altered flow dynamics.^65^^,^^66^ Based on this finding, we recommend screening for intracranial aneurysms with computed tomography angiography in all patients with ACCA and a hypoplastic or tortuous ipsilateral ICA.

Atherosclerotic stenosis of the ipsilateral ICA was less common, with severe stenosis observed in 6.3% of patients. Although this exceeds the 3.1% prevalence in the general population,^67^ the higher rate is likely due to selection bias, as symptomatic patients with ACCA are more likely to undergo imaging and be reported.

We report successful open surgical treatment for severe ipsilateral ICA stenosis in the setting of ACCA. Prior reports describe successful endovascular treatment in patients without significant ICA tortuosity or hypoplasia.^34^^,^^38^ In our case, the diseased ICA segment was notably tortuous and crossed anterior to the ECA, with potential for dynamic compression. We believe this anatomy made open surgical reconstruction a safer option than stenting in our case, with potentially lower long-term risk. We also felt that transposition of the ICA was lower risk than endarterectomy due to the proximal nature of the lesion. The clavicle was in very close proximity, which limited exposure and operative maneuverability.

Our approach involved transection and oversewing of the proximal ICA with transposition to the ECA, which was well-tolerated and effective. This technique has been described once previously, and in that case, was used to treat aneurysmal disease rather than stenosis.^59^ The safety of oversewing the proximal ICA during transposition is further supported by our case. Stenting is a reasonable revascularization option for patients with favorable anatomy and who are high risk for open surgery. Transcarotid artery revascularization (TCAR) is a hybrid revascularization strategy with strict anatomic inclusion criteria that has not been reported in ACCA. Theoretically ACCA alone does not pose contraindication to TCAR, and it should be safe and feasible in patients with normal ICA morphology who otherwise meet anatomic criteria. We chose not to perform TCAR in our case due to the proximal nature of the lesion.

Anatomically, some cadaveric studies have shown absence of the carotid body on the side of ACCA,^68^ but it remains unclear whether this has any clinical implications. Specifically, the risk of postendarterectomy hypertension or other baroreceptor-related effects in patients with ACCA has not been studied. Finally, the development of ICA atherosclerosis is theoretically less likely in ACCA due to the absence of a carotid bifurcation. Our data suggests that arterial tortuosity is likely a risk factor for development of atherosclerosis when the carotid bifurcation is absent. However, many studies we reviewed did not report the objective proximity of lesions relative to the aorta or relative to tortuous segments. Medical comorbidities predisposing to atherosclerosis were also seldom reported. We recommend future case reports and case series include this information when ACCA presents with severe stenosis of the ipsilateral ICA.

Our report has limitations. This review was limited to English-language articles and so some selection bias may exist due to excluding relevant articles in other languages. Gray literature sources were also not searched. We accounted for bias by including a critical appraisal tool in our methodology. There is significant reporting bias in the ACCA literature with respect to race, ethnicity, and clinical management of patients. It was often implied but not explicitly stated that asymptomatic patients underwent conservative management. Monitoring and screening strategies for carotid atherosclerosis were rarely discussed and longitudinal follow-up information beyond the initial diagnosis encounter was also underreported. Future reports of ACCA should adhere to quality-enhancing recommendations such as the Surgical Case Report (SCARE) guidelines.^69^

## Conclusion

ACCA frequently presents with cerebrovascular symptoms, including acute CVA or TIA in 33% of cases and is also associated with abnormal ICA morphology. Patients with ACCA and hypoplastic or tortuous ipsilateral ICA should undergo head imaging to evaluate for associated intracranial aneurysms. In cases of severe atherosclerotic disease of the ipsilateral ICA, carotid revascularization remains the standard of care. Both open and endovascular approaches are viable, with the choice of intervention primarily determined by arterial anatomy.

## Funding

None.

## Disclosures

None.

## Appendix (online only)

Supplementary Table I (online only)

Supplementary Table II (online only)

Supplementary Table III (online only)
