# Supplementary material for: Systematic review of clinical literature for absent common carotid artery
Source: J Vasc Surg Cases Innov Tech. 2025 Nov 12;12(1):102039. doi: 10.1016/j.jvscit.2025.102039 (PMC12712592; doi:10.1016/j.jvscit.2025.102039)
Supplement: Supplementary Table II (online only) [file mmc2.docx]

**Supplemental Table I:** Full results of systematic review

| Author | Year | Sex | Age (y) | Presentation | ACCA laterality | ICA stenosis | ICA hypoplasia or tortuosity | Aneurysm | Cerebrovascular treatment | Complication / mortality |
| --- | --- | --- | --- | --- | --- | --- | --- | --- | --- | --- |
| Hasio^5^ | 2025 | F | 63 | Incidental | R | No | H + T | No | No | No |
| Ramputi^2^ | 2024 | M | 61 | Incidental | R | No | No | No | No | No |
| Koester^6^ | 2024 | M | 59 | TIA | R | No | No | No | No | No |
| Tao^7^ | 2023 | M | < 1 | CHD workup | L | No | No | No | No | No |
| Park^8^ | 2023 | F | 10 | Other - Papilledema | L | No | H | No | No | No |
| Feleke^9^ | 2023 | F | 13 | Pulsatile neck mass | BL | No | No | No | No | No |
| Hiratsuka^10^ | 2023 | M | 42 | CVA | L | No | H + T | No | No | No |
| Yang^11^ | 2022 | M | 48 | Incidental | L | No | No | No | No | No |
| AlAni^12^ | 2022 | M | 35 | TIA | L | No | H | No | No | No |
| Cakirer^13^ | 2022 | F | 68 | TIA | L | No | No | No | No | No |
| Lee^14^ | 2021 | F | 39 | Other - Hearing loss | L | No | No | No | No | No |
| Sinha^15^ | 2020 | M | < 1 | CHD workup | L | No | No | No | No | No |
| Guarinello^16^ | 2020 | F | 50 | Other - R carotid bruit | R | No | No | No | No | No |
| Logan^17^ | 2020 | M | 78 | Screening | L | No | H + T | No | No | No |
| Davis^18^ | 2019 | M | 89 | TIA | L | Severe | No | No | No | No |
| Kwak^19^ | 2019 | F | 57 | CVA | L | No | H | AcommA, Basilar A | Aneurysm coiling | No |
| Hosn^20^ | 2018 | F | 70 | Other - L carotid bruit | R | Moderate | No | No | No | No |
| Uchino^21^ | 2018 | M | 45 | Other - Aortic dissection | R | No | No | No | No | No |
| Perez-Garcia^22^ | 2018 | F | 64 | TIA | L | No | H | No | No | No |
| Rawat^23^ | 2017 | M | 15 | Headache | BL | No | No | No | No | No |
| Quinones^24^ | 2017 | F | 28 | Headache | R | No | H | No | No | No |
| Braun^25^ | 2017 | F | 27 | Other - Trauma | L | No | No | No | No | No |
| Ghuman^26^ | 2017 | F | 30 | CVA | L | No | H + T | LICA | Craniotomy | No |
| Masri^27^ | 2016 | F | 90 | CVA | L | Severe | H | No | No | No |
| Bhat^28^ | 2016 | U | < 1 | CHD workup | L | No | No | No | No | No |
| Guha^29^ | 2016 | M | 8 | Pulsatile neck mass | R | No | No | No | No | No |
| Goyal^30^ | 2016 | M | 30 | Headache | L | U | U | No | No | No |
| Tahir^31^ | 2016 | M | 47 | Other - Neck pain | L | Yes | Yes | LICA | Open LICA aneurysm resection, end-to-end LICA anastomosis | No |
| Choi^32^ | 2015 | F | 65 | Vertigo | R | No | No | No | No | No |
| Ulger^33^ | 2014 | F | 64 | Vertigo | R | No | No | No | No | No |
| Berczi^34^ | 2014 | M | 72 | TIA | R | Severe | No | No | RICA stent | No |
| Malm^35^ | 2013 | M | 75 | Incidental | L | No | No | No | No | No |
| Kobayashi^36^ | 2013 | U | < 1 | CHD workup | BL | Absent ICA | Absent ICA | No | No | Death |
| Supsupin^37^ | 2012 | M | 25 | Incidental | R | No | No | No | No | No |
| Toyota^38^ | 2012 | M | 54 | CVA | R | Severe | T | No | RICA stent | No |
| Wood^39^ | 2011 | F, F, F | 77, 69, 51 | TIA, Incidental, Vertigo | R, R, R | Mild, Mild, No | No, No, No | No, No, No | No, No, No | No, No, No |
| Cao^40^ | 2011 | M | 42 | Vertigo | L | No | H | No | No | No |
| Drazin^41^ | 2010 | F | 41 | CVA | R | No | No | ACommA | Aneurysm coiling | No |
| Xie^42^ | 2010 | M | 42 | Steal - Subclavian | L | Absent ICA | Absent ICA | RICA | Craniotomy, RICA aneurysm clipping | No |
| Yim^43^ | 2009 | F | 52 | Headache | R | No | No | No | No | No |
| Cerase^44^ | 2009 | M | 33 | Incidental | R | No | H | No | No | No |
| Monaco^45^ | 2009 | F | 15 | CVA | R | No | No | No | Craniotomy | No |
| Cherian^46^ | 2008 | F | 18 | Pulsatile neck mass | L | No | No | No | No | No |
| Chen^47^ | 2008 | F | 53 | CVA | R | Absent ICA | Absent ICA | ACA | No | Death |
| Onbas^48^ | 2006 | M | < 1 | CHD workup | L | No | No | No | No | No |
| Purkayastha^49^ | 2006 | F | < 1 | CHD workup | L | No | No | No | No | Death |
| Sena^50^ | 2006 | M | 57 | Screening | R | No | No | No | No | No |
| Kocogullari^51^ | 2005 | F | 38 | Pulsatile neck mass | L | No | H | LICA | Open LICA aneurysm resection, end-to-end LICA anastomosis | No |
| Horowitz^52^ | 2003 | F | 50 | TIA | L | No | No | No | No | No |
| Maybody^53^ | 2003 | F | 72 | TIA | R | No | H + T | No | Contralateral LICA stent | No |
| Rossitti^54^ | 2001 | F | 40 | CVA | L | No | No | R carotid siphon, L sup. cerebellar A | No | No |
| Dahn^55^ | 1999 | F | 67 | Other – Facial numbness | L | No | H + T | No | No | No |
| Kjellin^56^ | 1999 | F | < 1 | Incidental | R | Absent ICA | Absent ICA | No | No | No |
| Warschewske^57^ | 1999 | F | 42 | Other – L homonymous hemianopsia | L | No | H + T | LICA | No | No |
| Woodruff^58^ | 1995 | F | 48 | Headache | L | No | No | No | No | No |
| Jerius^59^ | 1995 | M | M | Steal - Vertibrobasilar | R | Moderate | H | No | Open transposition RICA-to-RECA | No |
| Akduman^60^ | 1994 | F | 41 | Pulsatile neck mass | L | No | No | No | No | No |
| Kunishio^61^ | 1987 | M | 70 | CVA | L | Absent ICA | Absent ICA | ACA, MCA | Craniotomy, L MCA aneurysm clipping | No |
| Roberts^62^ | 1978 | F | 21 | CVA | BL | No | No | No | No | Death |
| Bryan^63^ | 1978 | F | 49 | TIA | L | No | H + T | No | No | No |
| Mullins^64^ | 1973 | F | < 1 | Pulsatile neck mass | R | No | No | No | No | No |
