# Supplementary material for: Systematic review of clinical literature for absent common carotid artery
Source: J Vasc Surg Cases Innov Tech. 2025 Nov 12;12(1):102039. doi: 10.1016/j.jvscit.2025.102039 (PMC12712592; doi:10.1016/j.jvscit.2025.102039)
Supplement: Supplementary Table III (online only) [file mmc3.docx]

**Supplemental Table III:** Critical appraisal of case report quality

| Author | Demographic characteristics | Patient history | Clinical condition | Diagnostic tests | Interventions or treatments | Post-intervention clinical condition | Adverse events or harms | Takeaway lessons | Total score |
| --- | --- | --- | --- | --- | --- | --- | --- | --- | --- |
| Hasio^5^ | U | Yes | Yes | Yes | Yes | Yes | Yes | Yes | 7 |
| Ramputi^2^ | U | Yes | Yes | Yes | Yes | Yes | Yes | Yes | 7 |
| Koester^6^ | Yes | Yes | Yes | Yes | U | U | U | Yes | 5 |
| Tao^7^ | U | Yes | Yes | Yes | U | Yes | U | No | 4 |
| Park^8^ | U | Yes | Yes | Yes | U | U | U | Yes | 4 |
| Feleke^9^ | U | Yes | Yes | Yes | Yes | Yes | Yes | Yes | 7 |
| Hiratsuka^10^ | U | Yes | Yes | Yes | U | U | U | Yes | 4 |
| Yang^11^ | U | Yes | Yes | Yes | Yes | Yes | Yes | Yes | 7 |
| AlAni^12^ | U | Yes | Yes | Yes | Yes | Yes | Yes | Yes | 7 |
| Cakirer^13^ | U | Yes | Yes | Yes | No | No | No | Yes | 4 |
| Lee^14^ | U | Yes | Yes | Yes | Yes | Yes | Yes | Yes | 7 |
| Sinha^15^ | U | U | No | Yes | Yes | Yes | Yes | No | 4 |
| Guarinello^16^ | U | Yes | Yes | Yes | Yes | Yes | Yes | Yes | 7 |
| Logan^17^ | U | U | No | Yes | No | No | No | No | 1 |
| Davis^18^ | U | Yes | U | Yes | No | No | No | No | 2 |
| Kwak^19^ | U | Yes | Yes | Yes | U | Yes | Yes | Yes | 6 |
| Hosn^20^ | U | Yes | Yes | Yes | Yes | Yes | Yes | Yes | 7 |
| Uchino^21^ | U | U | Yes | Yes | U | Yes | Yes | Yes | 5 |
| Perez-Garcia^22^ | U | U | Yes | Yes | Yes | Yes | Yes | Yes | 6 |
| Rawat^23^ | U | U | No | Yes | Yes | Yes | Yes | No | 4 |
| Quinones^24^ | U | Yes | Yes | Yes | Yes | Yes | No | Yes | 6 |
| Braun^25^ | U | U | U | Yes | Yes | No | Yes | No | 3 |
| Ghuman^26^ | U | U | U | Yes | Yes | Yes | Yes | Yes | 5 |
| Masri^27^ | U | Yes | Yes | Yes | U | U | U | No | 3 |
| Bhat^28^ | U | Yes | Yes | Yes | Yes | Yes | Yes | Yes | 7 |
| Guha^29^ | U | Yes | Yes | Yes | Yes | Yes | Yes | Yes | 7 |
| Goyal^30^ | U | Yes | Yes | Yes | U | U | U | Yes | 4 |
| Tahir^31^ | U | Yes | Yes | Yes | Yes | Yes | Yes | Yes | 7 |
| Choi^32^ | U | Yes | Yes | Yes | U | U | U | U | 3 |
| Ulger^33^ | U | U | Yes | Yes | Yes | Yes | Yes | Yes | 6 |
| Berczi^34^ | U | Yes | Yes | Yes | Yes | Yes | Yes | Yes | 7 |
| Malm^35^ | U | Yes | Yes | Yes | Yes | Yes | Yes | Yes | 7 |
| Kobayashi^36^ | U | U | U | Yes | U | Yes | Yes | No | 3 |
| Supsupin^37^ | U | U | U | Yes | Yes | Yes | Yes | Yes | 5 |
| Toyota^38^ | U | Yes | U | Yes | Yes | Yes | Yes | Yes | 6 |
| Wood^39^ | U | U | U | Yes | U | U | U | Yes | 2 |
| Cao^40^ | U | Yes | Yes | Yes | Yes | Yes | Yes | Yes | 7 |
| Drazin^41^ | U | Yes | Yes | Yes | Yes | Yes | Yes | Yes | 7 |
| Xie^42^ | U | Yes | Yes | Yes | Yes | U | U | Yes | 5 |
| Yim^43^ | U | Yes | Yes | Yes | U | U | U | U | 3 |
| Cerase^44^ | Yes | Yes | Yes | Yes | Yes | Yes | Yes | Yes | 8 |
| MoYesco^45^ | U | Yes | Yes | Yes | Yes | No | No | Yes | 5 |
| Cherian^46^ | U | Yes | Yes | Yes | Yes | Yes | Yes | Yes | 7 |
| Chen^47^ | U | U | Yes | Yes | Yes | Yes | Yes | Yes | 6 |
| Onbas^48^ | U | U | Yes | Yes | U | U | U | Yes | 3 |
| Purkayastha^49^ | U | Yes | Yes | Yes | Yes | Yes | Yes | Yes | 7 |
| Sena^50^ | Yes | U | Yes | Yes | Yes | Yes | Yes | U | 6 |
| Kocogullari^51^ | U | U | Yes | Yes | Yes | Yes | Yes | Yes | 6 |
| Horowitz^52^ | U | U | U | Yes | U | U | U | Yes | 2 |
| Maybody^53^ | U | Yes | U | Yes | Yes | Yes | Yes | Yes | 6 |
| Rossitti^54^ | U | U | U | Yes | U | U | U | Yes | 2 |
| Dahn^55^ | U | Yes | Yes | Yes | Yes | Yes | Yes | Yes | 7 |
| Kjellin^56^ | U | Yes | Yes | Yes | Yes | Yes | Yes | Yes | 7 |
| Warschewske^57^ | U | U | Yes | Yes | U | U | U | Yes | 3 |
| Woodruff^58^ | U | U | Yes | Yes | Yes | Yes | Yes | U | 5 |
| Jerius^59^ | U | Yes | Yes | Yes | Yes | Yes | Yes | Yes | 7 |
| Akduman^60^ | U | U | Yes | Yes | Yes | U | U | Yes | 4 |
| Kunishio^61^ | U | U | U | Yes | Yes | U | U | Yes | 3 |
| Roberts^62^ | U | U | Yes | Yes | Yes | Yes | Yes | Yes | 6 |
| Bryan^63^ | Yes | Yes | Yes | Yes | Yes | U | U | Yes | 6 |
| Mullins^64^ | U | Yes | Yes | Yes | Yes | U | U | Yes | 5 |
